# Current Approach in the Diagnosis and Management of Allergic Bronchopulmonary Aspergillosis in Children With Cystic Fibrosis

**DOI:** 10.3389/fped.2020.582964

**Published:** 2020-10-20

**Authors:** Birce Sunman, Dilber Ademhan Tural, Beste Ozsezen, Nagehan Emiralioglu, Ebru Yalcin, Uğur Özçelik

**Affiliations:** Department of Pediatric Pulmonology, Hacettepe University Faculty of Medicine, Ankara, Turkey

**Keywords:** ABPA, allergic bronchopulmonary aspergillosis, aspergillus, cystic fibrosis, children

## Abstract

Allergic bronchopulmonary aspergillosis (ABPA) is a complex pulmonary disorder characterized by a hypersensitivity reaction to *Aspergillus fumigatus*, and almost always seen in patients with cystic fibrosis (CF) and asthma. Fungal hyphae leads to an ongoing inflammation in the airways that may result in bronchiectasis, fibrosis, and eventually loss of lung function. Despite the fact that ABPA is thought to be more prevalent in CF than in asthma, the literature on ABPA in CF is more limited. The diagnosis is challenging and may be delayed because it is made based on a combination of clinical features, and radiologic and immunologic findings. With clinical deterioration of a patient with CF, ABPA is important to be kept in mind because clinical manifestations mimic pulmonary exacerbations of CF. Early diagnosis and appropriate treatment are important in preventing complications related to ABPA. Treatment modalities involve the use of anti-inflammatory agents to suppress the immune hyperreactivity and the use of antifungal agents to reduce fungal burden. Recently, in an effort to treat refractory patients or to reduce adverse effects of steroids, other treatment options such as monoclonal antibodies have started to be used. Intensive research of these new agents in the treatment of children is being conducted to address insufficient data.

## Introduction

*Aspergillus fumigatus* (*A. fumigatus*) is the most common ubiquitous airborne fungus, which causes allergic bronchopulmonary aspergillosis (ABPA) (1). Aspergillus spores are found in high concentrations in nature, especially in fertile soil, decaying vegetation, swimming pool water, leaky basements, bedding, and dust from homes (2). Hypersensitivity reactions that occur because of *A. fumigatus* allergens are allergic asthma, hypersensitivity pneumonia, and ABPA (3), which is a localized hypersensitivity reaction to the lung that develops against aspergillus antigens in the colonized bronchial mucus.

The prevalence of ABPA changes according to the population (child/adult), geographic region, or diagnostic criteria that have been used. At the same time, ABPA is believed to be underdiagnosed, especially in developing countries, because its clinical features are much the same as cystic fibrosis (CF). In asthmatic patients the prevalence is reported to be about 1 to 2% and is more common in adults than in children (4, 5). The prevalence is higher in CF patients than in asthmatic patients and thought to be 8.9% (ranged from 3 to 25%) with a significantly higher occurrence among adults (6, 7).

In this review, immunopathogenesis, clinical features, diagnosis, and current treatment modalities have been tried to be summarized. Although this review is based on the studies and case reports with the pediatric age group, some studies in adults and asthmatics have also been mentioned due to limited number of publications in children. By the way the diagnosis and treatment in children are not much different from adults and the treatment in CF is similar to asthmatics (8).

## Immunopathogenesis of ABPA

The pathogenesis of ABPA involves many immunologic reactions. These are Aspergillus-specific immunoglobulin (Ig)-E–mediated hypersensitivity, IgG-mediated immune complex hypersensitivity, and abnormal cell-mediated immune response (9). These hypersensitivity responses cause mucus impaction in the bronchi and bronchioles, as well as inflammatory cell infiltration in bronchial walls and peribronchial tissues. All of these reactions cause bronchiectasis and bronchocentric non-caseating granulomatosis (10, 11).

*Aspergillus conidia*, because of its small diameter (2–3 mm), can easily reach the pulmonary alveoli and deposit there. Once they reach the alveoli, spores germinate to produce fungal hyphae and continuously grow in the airways of patients with ABPA. *A. fumigatus* has several virulence factors to escape from the immune system including superoxide dismutases, catalases, mannitol, proteases, ribotoxin, phythiotic acid, phospholipases, gliotoxin, and hemolysin. Most of these proteins are known to be antigenic and are believed to be responsible for the immune response in ABPA. These virulence factors also damage the airway epithelium and cause a larger dose of antigenic factors to pass to the interstitial and vascular compartments. Antigenic cells with human leukocyte antigen (HLA)-DR5 or HLA-DR2 process these antigens together and present them to T lymphocytes in bronchoalveolar lymphoid tissue. In normal hosts, while the organism is eradicated with the T helper (Th)1 response, in patients with ABPA, an extreme Th2 response to the aspergillus antigen is seen, even if the Th1 response is not defective. Protease and antigen released by spores and hyphae cause activation of the innate immune system, and damage in the bronchial epithelium, which causes bronchiectasis and impaired mucociliary clearance. As a result, various chemokines including thymus and activation regulated chemokine (TARC), monocyte chemotactic protein 1, eotaxin, RANTES (regulated on activation, normal T-cell expressed, and secreted), interleukin (IL)-8, and macrophage inflammatory protein 1a are released in the airways. These cytokines activate the Th2 response and this causes the proliferation of CD4+ Th2 lymphocytes, which produce IL-4, IL-5, IL-9, IL-10, IL-13, and eosinophilic growth and survival, mast cell proliferation, IgG and IgE isotype switching occurs (9, 10).

Similarly in patients with CF, due to abnormal mucociliary clearence of secretions and defective innate immune responses, exposure to *A. fumigatus* spores results in accumulation and persistence of fungal spores within the smaller airways (12). Release of antigens, cytokins, and other virulence factors cause airway epithelial damage and antigenic factors are transmitted to the interstitial and vascular compartments (13). The immune response to ABPA in CF patients is also IL-4–mediated T helper cell (Th) type 2–predominant response, which is shown by CFTR mutant mouse expression profiling studies (14, 15).

Finally, there are some opinions about why some patients with CF develop ABPA. One of these is that the predominant CD4+ Th2 cell response can be related to genetic factors and this can explain why some patients with CF develop ABPA while others do not (16). Another conviction is that because patients with ABPA have an exaggerated response to IL-4 and produce a large amount of IgE, IgG, and IgA antibodies against *A. fumigatus* antigens, a gain of function polymorphism in the IL-4 receptor-α chain may be responsible for this situation (17). Lastly, some authors suggest that HLA-DRB1^*^1501 and HLA-DRB1^*^1503 confer the highest risk of developing ABPA, whereas HLA-DQ2 (HLA-DQB1^*^0201 in particular) provides relative protection against the development of ABPA (18–20).

Therefore, a combination of all these factors may determine the outcome of ABPA in patients with CF.

## Clinical Features

ABPA symptoms are usually non-specific and resemble clinical findings in CF. One-third of patients with CF are asymptomatic and are diagnosed as having ABPA in routine follow-up (7). The most common clinical findings are chronic productive cough and wheezing. Other symptoms are pleuritic chest pain and blood-stained sputum. Expectoration of golden-brownish mucus plugs is a characteristic finding in ABPA and is found in half of all patients (5, 21, 22). The dark mucus plugs are due to the increased production of tenacious mucus in the respiratory tract and consist of inflammatory cells including eosinophils, desquamated epithelial cells, and mucin (23, 24). Hemoptysis can occur due to severe airway inflammation and bronchiectasis (3). Constitutional symptoms such as low-grade fever, myalgia, and weight loss are found in 26% of patients with CF (25, 26). Physical examination is usually not noticeable except for crackles, rhonchi unresponsive to bronchodilator treatment, absence of respiratory sounds distal to dark mucus plugs, and clubbing. In end-stage disease, cor pulmonale findings may be present (4). ABPA should be suspected in a patient with CF who develops wheezing or major reductions of forced expiratory volume in one second (FEV1) without evidence of a CF exacerbation, that do not respond to appropriate antibiotics, standard physiotherapy and which is not explained by another etiology (3).

### Stages

The disease can be clinically divided into five stages as shown in Table 1 (2). Patients may be detected at any stage at the time of diagnosis and the transition from one stage to another may not be in order. It is also important to notify that ABPA serology is most likely to be positive in stage 1 and 3. Early diagnosis and treatment prevent the disease from progressing to stage 5.

**Table 1:** Clinical and serological characteristics of ABPA stages.

	**Definition**	**Total IgE**	**Precipitins**	**Eosinophilia**	**IgE- *A. fumigatus***	**IgG- *A. fumigatus***	**Lung infiltrates**
Stage 1 (acute stage)	The patient has all the clinical and radiologic features of ABPA, responds well to oral corticosteroid therapy, and corticosteroids can be discontinued. Patient is considered in remission if improvement continues for six months	+++	+	+	+	+	+
Stage 2 (remission)	At this stage, clinical and radiologic improvement is achieved. Total IgE is at least 25% decreased. Some patients may enter remission spontaneously. Stage 2 can persist indefinitely or the disease may recur	+	±	−	±	±	−
Stage 3 (relapse)	It has all the features of stage 1. If a patient is on routine follow-up, at least a doubling in serum IgE level with new infiltrations on chest radiography is seen	+++	+	+	+	+	+
Stage 4 (steroid-dependent stage)	The patient receives long-term high-dose systemic steroid therapy. When the steroid dose is tried to be reduced and stopped, it relapses	++	±	±	±	±	−
Stage 5 (end-stage lung disease)	Diffuse bronchiectasis, fibrosis, cor pulmonale has developed. Serum total IgE level can be normal or elevated	+	±	−	±	±	−

*ABPA, allergic bronchopulmonary aspergillosis; A. fumigatus, Aspergillus fumigatus, IgE, immunoglobulin E, IgG, immunoglobulin G*.

## Diagnosis

The diagnosis of ABPA in patients with CF is challenging and may be delayed because many of the diagnostic criteria crossover with the clinical manifestations of CF. There is no fully covered individual test that demonstrates the diagnosis of ABPA in patients with CF. The diagnosis is made through a combination of clinical characteristics, and radiologic and immunologic findings (27). There are different sets of diagnostic criteria for the diagnosis of ABPA in patients with CF. The diagnostic criteria are summarized in Table 2 according to historical date (28–31).

**Table 2:** History of acceptable criteria for diagnosing ABPA.

*Epidemiologic Study of Cystic Fibrosis* recommendations, 1999
Two of the following 3 criteria are required
• Immediate cutaneous reactivity to *A. fumigatus* • Precipitating antibodies to *A. fumigatus* • Serum total IgE of >1,000 IU/mL.
In addition, at least 2 of the following are required
• Bronchoconstriction • Peripheral blood eosinophil count >1,000 mL • History of pulmonary infiltrates • Elevated serum anti-*A. fumigatus* IgE or IgG •*A. fumigatus* in sputum found by smear or culture • Response to steroids treatment
*The UK Cystic Fibrosis Trust* recommendations, 2002
• Asthma symptoms • New chest radiography changes • Serum total IgE >500 IU/mL or four-fold increase in IgE titers • Raised specific IgE aspergillus RAST or positive skin prick test to *A. fumigatus* • Blood eosinophil count >500/mm^3^ • Positive Aspergillus culture in sputum or fungal hyphae
*Cystic Fibrosis Foundation* Consensus Conference recommendations, 2003
Classic case
• Acute or subacute clinical deterioration that is not attributable to another etiology • A serum total IgE level of >1,000 IU/mL (unless patient is receiving systemic steroids) • Presence of IgE antibodies to *A. fumigatus in vitro* or immediate cutaneous hypersensitivity to Aspergillus (skin test >3 mm) • Precipitating antibodies to *A. fumigatus* or serum IgG antibody to *A. fumigatus* by an *in vitro* test • New or recent abnormalities on chest radiography or computed tomography that do not respond to antibiotics and standard physiotherapy
Minimal diagnostic criteria
• Acute or subacute clinical deterioration that is not attributable to another etiology • A serum total IgE level of >500 IU/mL (unless patient is receiving systemic steroids) • Immediate cutaneous hypersensitivity to Aspergillus (skin test >3 mm) or presence of IgE antibodies to *A. Fumigatus*
Plus one of the following
• Precipitins to *A. fumigatus* or IgG antibody to *A. fumigatus in vitro* • New or recent infiltrates (or mucus plugging) on chest radiography or computed tomography that do not respond to antibiotics and standard physiotherapy
*American Academy of Allergy, Asthma & Immunology* Committee Report, 2012
Minimum essential criteria
• Asthma or cystic fibrosis • Immediate cutaneous reactivity on skin-prick testing • Elevated serum total IgE level (>1,000 ng/mL)
Plus one or both of the following
• Elevated serum IgE and IgG levels to *A. fumigatus* (at least twice asthma controls) • Proximal (central) bronchiectasis on radiography (inner two-thirds of lung on computed tomography)
*International Society for Human and Animal Mycology* Working Group, 2013
*Predisposing conditions*
Asthma or cystic fibrosis
*Obligatory criteria* (both must be present)
• Aspergillus skin test positivity or elevated IgE levels against *A. fumigatus* • Elevated total IgE concentration (typically >1,000 IU/mL)
*Other criteria* (at least 2 must be present)
• Precipitating serum antibodies to *A. fumigatus* or elevated serum Aspergillus IgG by immunoassay • Radiographic pulmonary opacities consistent with ABPA • Total eosinophil count of >500 cells/mL in patients who are steroid naive (may be historical)

*ABPA, allergic bronchopulmonary aspergillosis; A. fumigatus, Aspergillus fumigatus; IgE, immunoglobulin E; IgG, immunoglobulin; RAST, radioallergosorbent test*.

According to Cystic Fibrosis Foundation Consensus Conference recommendations (2003), which is the most common used definition for diagnosis (3):

### Classic Case

1. Clinical deterioration, acute or subacute, which is unexplained by another etiology;
2. Serum total IgE concentration of >1,000 IU·mL−1 (unless the patient is receiving systemic corticosteroids);
3. Immediate cutaneous reactivity (skin prick test wheal >3 mm in diameter with surrounding erythema) to Aspergillus or *in vitro* presence of serum IgE antibody to A. fumigatus;
4. Precipitating antibodies to A. fumigatus or serum IgG antibody to A. fumigatus in an *in vitro* test;
5. New or recent abnormalities on chest radiography (infiltrates or mucus plugging) or chest computed tomography (CT) (bronchiectasis) that do not respond to appropriate antibiotics and standard physiotherapy.

### Minimal Diagnostic Criteria

1. Clinical deterioration, acute or subacute, which is unexplained by another etiology;
2. Serum total IgE concentration of >500 IU·mL−1. If total IgE level is 200–500 and ABPA is suspected, repeat testing in 1–3 months;
3. Immediate cutaneous reactivity to Aspergillus (skin prick test wheal >3 mm in diameter with surrounding erythema while the patient is not being treated with systemic antihistamines) or *in vitro* presence of IgE antibody to A. fumigatus;
4. One of the following: (a) precipitins to A. fumigatus or *in vitro* demonstration of IgG antibody to A. fumigatus; or (b) new or recent abnormalities on chest radiography (infiltrates or mucus plugging) or chest CT (bronchiectasis) that do not respond to appropriate antibiotics and standard physiotherapy.

The suggestions of Cystic Fibrosis Foundation Consensus Conference for screening ABPA in CF (3):

1. Patients >6 years of age should be considered suspicious for ABPA.
2. Patients should be checked for serum total IgE concentration annually. If the serum total IgE concentration is >500 IU·mL−1, get immediate cutaneous reactivity to A. fumigatus or use an *in vitro* test for IgE antibody to A. fumigatus. If results are positive, consider diagnosis on the basis of minimal criteria;
3. If the serum total IgE concentration is 200–500 IU·mL−1, repeat the test in 1–3 months if there is a suspicion for ABPA.

**Figure 1:**
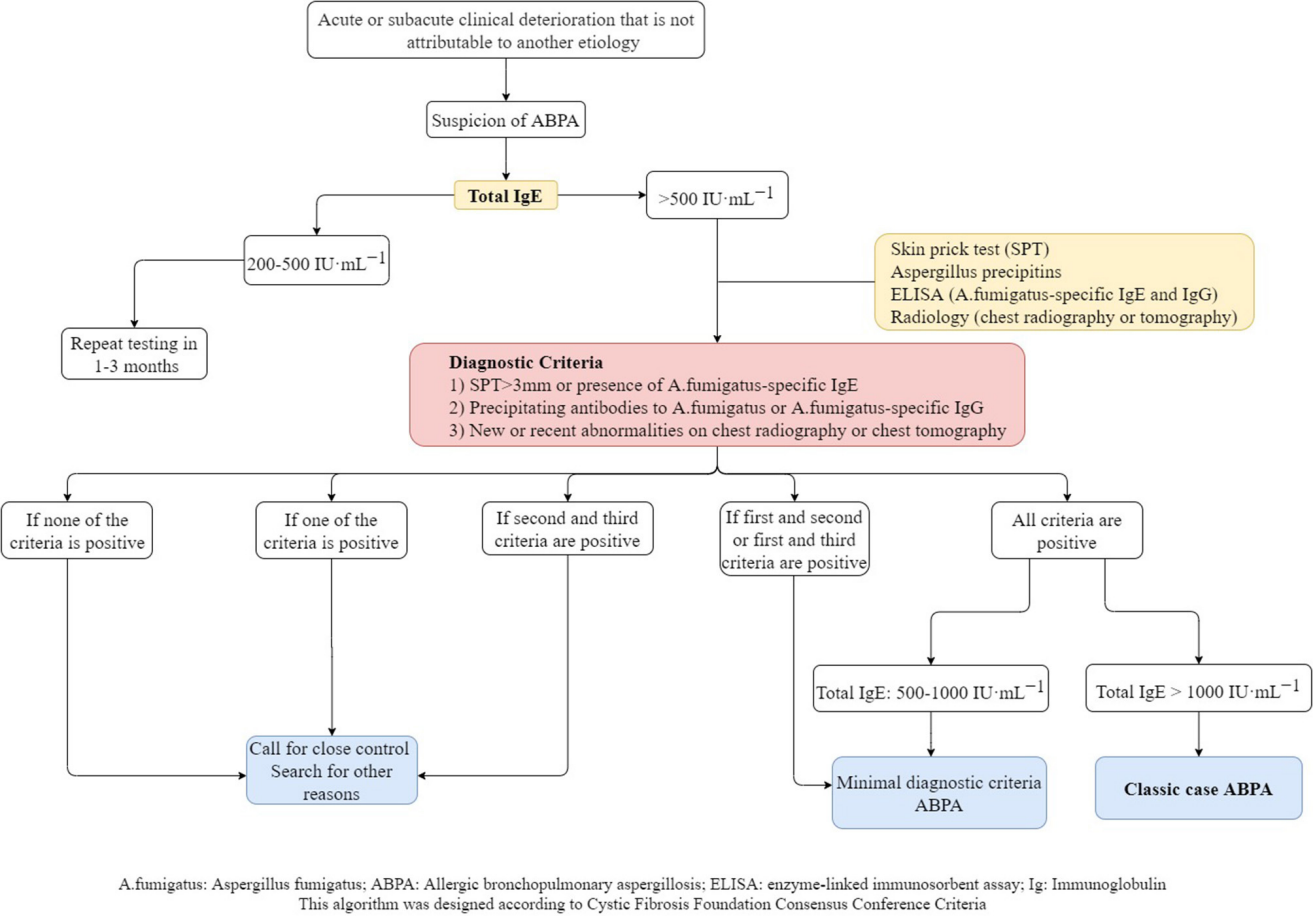
Diagnostic algorithm of ABPA in CF.

The diagnostic algorithm created with the criteria suggested by the Cystic Fibrosis Foundation is showed in Figure 1.

Although there are many diagnostic criteria for ABPA, Maleki et al. showed that there was no significant differences on the reported rate of ABPA prevelance between the Cystic Fibrosis Foundation and International Society for Human and Animal Mycology (ISHAM) diagnostic criteria (32).

### Clinical Findings

Acute/subacute clinical worsening defined as cough, increased amount of sputum or changing in color of sputum, wheeze, dyspnea, the onset of new fever, weight loss, exercise-induced asthma and decrease in pulmonary function, that does not respond to appropriate treatment and is not explained with another etiology (3).

### Serum Total IgE

This can be used for the detection of fungal sensitization (33). Values of total IgE as high as >500 IU·mL^−1^ (34) or even >1,000 IU·mL^−1^ (35) or >2-fold rise from baseline total IgE have been suggested as diagnostic markers unless the patient is receiving systemic corticosteroids. If the patient is using systemic steroids, retesting is recommended after the completion of steroid treatment (3). Irregular changes in IgE values with clinical symptoms can be a marker for exacerbations and responses to therapy. In patients with ABPA, despite total serum IgE levels often being in concordance with clinical activity and treatment, it is not sufficiently specific for the diagnosis (29, 36, 37).

### Aspergillus Skin Test

Another investigation for the detection of sensitization to *A. fumigatus* is the Aspergillus skin test, which shows immediate cutaneous hypersensitivity to *A. fumigatus* (38). However, it shows heterogenity among different centers in terms of procedures, interpretation, and the use of different commercial fungal preparations (39). A skin prick test should be performed for Aspergillus skin testing; if the results are negative it should be confirmed by an intradermal test. Intradermal skin tests are usually preferred for the diagnosis of Aspergillus sensitization because they are more sensitive than the skin prick test (40).

Both type I (immediate) and type III (delayed) skin sensitivity with different Aspergillus antigens can be positive in patients with ABPA. Although type III responses are mainly suppressed by steroid treatment, there is little or no effect of steroids on the type I reactions. For the wheal of immediate skin sensitivity, the authorities have suggested a diameter of surrounding erythema >3 mm to be considered as a positive result (3). Aspergillus skin test is sensitive enough that the lack of a positive skin test reduces the likelihood of ABPA diagnosis; however, the specificity of the test is moderately low, which means it can be positive in patients with CF without ABPA (41). Therefore, a positive Aspergillu*s* skin test must always be followed up with serologic and radiologic testing to confirm ABPA.

### Serum Specific IgE to *A. fumigatus*

This is not an appropriate biomarker and lacks sufficient specificity for ABPA because nearly 35% to 50% of patients with CF demonstrate detectable sensitization to *A. fumigatus* despite not having ABPA. However, elevated specific IgE to *A. fumigatus* is a more sensitive marker than total IgE like the aspergillus skin test for ABPA in CF (38, 39, 42, 43). In 2013, ISHAM accepted 0.35 kUA·L^−1^ as a cut-off value for anti-Aspergillus sensitization and this value was changed and accepted as 0.10 kUA·L^−1^ by the US Food and Drug Administration (FDA) in 2008 and recommended by a 2015 consensus guideline (29, 39). The level of specific IgE to *A. fumigatus* can act as a marker of an exacerbation or remission like total IgE.

### Precipitating Antibodies or Serum IgG Antibody to *A. fumigatus*

There are few methods to evaluate the *A. fumigatus* precipitating antibody or specific IgG to the crude antigen. These antibodies, have been found to be a sensitive sign for ABPA in patients with CF. Precipitating antibodies to *A. fumigatus* are usually in the IgG isotype, in particular of the IgG1, IgG2, and IgG4 subclasses, and their increased levels have been reported in patients with CF and ABPA (44, 45).

Traditionally, IgG antibodies against *A. fumigatus* were measured by immunoprecipitation and counterimmunoelectrophoresis (CIE) using double gel diffusion techniques and called *Aspergillus* precipitins (46–48). Because of poor sensitivity and subjective qualitative results of these methods, commercial ImmunoCAP systems using enzyme-linked immunosorbant assays (ELISA) have been developed and this method has increased the detection of cases with ABPA. Recently, this commercial system is the most widely used method for quantifying specific IgG. The results of few studies also suggest that *A. fumigatus* specific IgG measured by the ImmunoCAP system is more sensitive than *Aspergillus* precipitins measured by the double diffusion method (47, 48). The best cutoff value for *A. fumigatus* specific IgG using ImmunoCap system has been reported as 26.9 mgA/L with 88% sensitivity and 100% specificity. Although the sensitivity of *A. fumigatus* specific IgG detected by double gel diffusion technique has been reported low as 27%; the sensitivity of commercial ImmunoCAP systems was 89% in the diagnosis of ABPA (47). However, the cutoff values have been varied in patients with CF within different studies (47–50). The prevalence of serum IgG antibodies to *A. fumigatus* has been reported to increase with age in patients with CF, regardless of ABPA (51). *Aspergillus* precipitins may represent previous exposure, but high levels of precipitins may indicate increased probability of ABPA (31). *A. fumigatus-*specific IgG levels may suggest disease activity, which can be evidenced by radiographic changes and clinical exacerbations, whereas serum precipitins do not reflect disease activity in most cases (1).

### Peripheral Blood Eosinophilia

Peripheral blood eosinophil counts of >1,000 cell/ μL were previously considered as a major criteria for the diagnosis of ABPA (52, 53). However, now it is known to be of limited value in diagnosing patients with CF and ABPA because high eosinophil counts may be present due to chronic *Pseudomonas aeruginosa* infection (3) and many other disorders other than ABPA (54, 55). In a recent study on children with CF reported that 75% of ABPA patients had eosinophil count >400 cells/μL and 40% of them having counts >1,000 cells/μL, while none of the patients in the *A. fumigatus* sensitized and non-sensitized groups had eosinophilia and these findings suggested that eosinophil count could be a specific biomarker for ABPA in children (56).

### New Serologic Tests

#### Specific IgE Antibodies Against Recombinant A. fumigatus Allergens

Recombinant allergens are raw extracts from *A. fumigatus* which uses for immunological assays of ABPA. There are recognized 23 specific allergens of *A. fumigatus* but five of them (rAsp f1, f2, f3, f4, and f6) are commercially available (57). However, those allergens can cross-react with other fungal antigens. Due to rAsp f1 and f2 have minimal cross-reactivity with other fungal antigens, they are considered as the specific allergens for *A. fumigatus* (58). Specific IgE against a combination of rAsp f1 or f3 found to be the most sensitive (97%) and specific IgE against a combination of rAsp f4 or f6 had highest specificity (99%) for diagnosing ABPA in patients with asthma (57). Some authors suggested combining serum total IgE with specific IgE to recombinant *A. fumigatus* allergen (rAspf) to differentiate ABPA from sensitization (59). A recent study reported that IgE against rAsp f1 and f2 were found to be the most useful in differentiating ABPA from *A. fumigatus* sensitization in patients with asthma (60).

#### Thymus and Activation-Regulated Chemokine (TARC)

TARC is produced as a result of the antifungal immune response. The serum levels of TARC are found to be increased in patients with CF and ABPA (61). TARC was found to be a more sensitive and specific marker of ABPA when it was compared with other serum markers. TARC levels are increased even before the development of clinical features of ABPA and before total IgE increment (59). TARC stays elevated for a prolonged period of time; therefore, the changes in TARC levels can be a sign of exacerbations and remissions of ABPA (59, 61). However, it has not been added to classic case definitions.

#### Cellular Allergen Stimulation Test (CAST)

CAST measures cysteinyl-leukotrienes, which are produced *in vitro* by allergen-stimulated basophils. CAST is used for the diagnosis of allergic and pseudoallergic reactions. CAST has a high sensitivity (100%) but a low specificity (74%) for ABPA. The combination of a positive CAST, serum total IgE >500 IU·mL^−1^, and positive IgE antibodies against rAsp f4 and f6 was found only in those with ABPA, giving rise to 100% specificity (62).

Further studies with a larger number of patients are required to investigate CAST and TARC before they become routine investigations for ABPA.

#### Basophil Activation Test (BAT)

BAT is an *in vitro* flow cytometry-based cellular assay that measures the activation of basophils with Ig-E mediated mechanism and using stimulation with *A. fumigatus* extract and CD63, CD193, and CD203c as activation surface markers which they found to have high diagnostic accuracy for ABPA. This test should be performed within the four hour of blood collection to increase viability and functionality of basophils; because basophil reactivity decreases over time (63). Different studies suggest that BAT is a useful, reliable diagnostic tool especially for ABPA in patients with CF (64, 65) and it is useful in distinguishing ABPA from *Aspergillus* colonization and sensitization in patients with CF (66, 67) but not in patients with asthma (68). However, BAT needs a flow cytometer and the requirement of performing this test immediately after the collection of blood sample limit its usability.

### Culture of Sputum for *A. fumigatus*

Sputum culture is a supportive marker for ABPA (43, 69), whereas others have considered it as only a minor criterion because *A. fumigatus* hyphae in sputum smears or *A. fumigatus* in sputum cultures may not be detected in ABPA or can also be seen in other pulmonary diseases (70, 71). In ABPA, rates of culture positivity were reported as 40% to 60% in different studies (72). Also, *A. fumigatus* culture-negative patients with ABPA were found to have *A. fumigatus* DNA in their sputum (73). Aspergillus polymerase chain reaction (PCR) is more sensitive than culture in ABPA diagnosis and it may be used to monitor the efficacy of antifungal therapy (72). Both real-time Aspergillus PCR and galactomannan in respiratory samples have also been used for enhanced recognition of ABPA in CF with traditional immunological tests (serum total IgE, *A. fumigatus*-specific IgE and IgG) (74).

### Pulmonary Function Tests

In mild or early stages of ABPA, partially reversible airflow obstruction is a common pulmonary function test finding. Prolonged airflow obstruction and decreased lung volumes in total lung capacity (TLC), vital capacity (VC), and FEV1 suggest interstitial changes in progressive disease (75, 76). The diffusing capacity of lung for carbon monoxide (DLCO) may be decreased during an exacerbation and it remains low at the end stage of ABPA (5). Despite pulmonary function tests not being diagnostic for ABPA, they are useful during follow-up to monitor improvement.

### Bronchoscopy

Bronchoscopic evaluation and histology are not necessary for the diagnosis of ABPA and bronchoscopy may be performed in patients with ABPA when the diagnosis is unclear. Bronchoalveolar lavage (BAL) shows elevated levels of IgA, IgG, IgM, and IgE, as well as elevated eosinophil counts. However, the sensitivity of staining BAL washes or sputum samples for Aspergillus is poor. Detecting Aspergillus species in BAL is not specific for active disease of ABPA because it may reflect colonization (75, 77).

Interpretation of diagnostic findings of ABPA are summarized in Table 3.

**Table 3:** Interpretation of diagnostic findings of ABPA.

**Investigation**	**Result**	**Interpretation**
Serum total IgE levels	Normal	Exclude ABPA
	>500–1,000 IU/mL	Consider ABPA
Aspergillus skin test	Type 1 reaction	ABPA characteristic
	Type 3 reaction	ABPA characteristic, Immune complex hypersensitivity reaction, suggest fungal hypersensitivity
Serum specific IgE to *A. fumigatus*	Elevated IgE levels	Consider ABPA or Aspergillus hypersensitivity
Serum precipitins (IgG) against *A. fumigatus*	IgG antibodies present	Supportive, not diagnostic
Peripheral eosinophilia	Elevated	Supportive, not diagnostic
Sputum culture	Presence of *A. fumigatus*	Supportive of ABPA Seen in ≤ 50% patients
Aspergillus PCR	Presence of Aspergillus DNA	Supportive, not diagnostic Consider also Aspergillus hypersensitivity, Aspergillus bronchitis, colonization
Pulmonary function tests	Typical obstructive findings	No role in diagnosis Can assess severity, improvement
Bronchoscopy	Elevated eosinophil count and levels of IgA, IgG, IgM, and IgE	Unclear for ABPA diagnosis Not necessary for ABPA diagnosis

*ABPA, allergic bronchopulmonary aspergillosis; A. fumigatus, Aspergillus fumigatus; Ig, immunoglobulin; DNA, deoxyribonucleic acid; PCR, polymerase chain reaction*.

### Radiologic Manifestations of ABPA

Both chest radiography and chest CT are useful for the diagnosis of ABPA. Radiologic findings are summarized in Table 4.

**Table 4:** Radiologic findings in ABPA.

**Chest radiography**	**Computed tomography**
*Transient changes* **Common** Patchy areas of consolidation Radiologic infiltrates, due to mucoid impaction in dilated bronchi; • Toothpaste shadows • Gloved finger shadows Collapse; segmental or lobar **Uncommon** Bronchial wall thickening; • Tramline shadows Air-fluid levels from dilated central bronchi filled with fluid Perihilar infiltrates simulating adenopathy Massive consolidation: unilateral or bilateral Small nodules Pleural effusions *Permanent changes* **Common** Parallel-line shadows representing bronchial widening Ring-shadows 1–2 cm in diameter representing dilated bronchi en face Pulmonary fibrosis: fibrotic scarred upper lobes with cavitation **Uncommon** Pleural thickening Mycetoma formation Linear scars	**Common** Central bronchiectasis Mucus plugging with bronchoceles Consolidation Non-homogeneous patchy opacities Centrilobular nodules with tree-in-bud opacities Bronchial wall thickening Areas of atelectasis Cavitation Mosaic perfusion with air trapping on expiration **Uncommon** High-attenuation mucus (most helpful finding in differential diagnosis) Pleural involvement Randomly scattered nodular opacities

*ABPA, allergic bronchopulmonary aspergillosis*.

Chest radiography has 50% sensitivity for the diagnosis of ABPA. It can be normal in the early stages but temporary or permanent parenchymal opacities can also be seen. Parenchymal infiltrate and bronchiectasis are mostly in the upper lobes; however, all lobes may be affected (40). Central bronchiectasis is one of the hallmarks of ABPA, and bronchiectasis affecting more than three lobes is highly suggestive of diagnosis (78, 79). A massive homogeneous shadow without fissure displacement usually located in the upper and middle lobes that frequently shifts from one side to another is the most common abnormality seen on a chest radiography with ABPA. The shadow may be patchy, triangular, oblong, or lobar (6). “Ring sign” indicating bronchial inflammation with or without plugs can be a sign of bronchial wall thickening or bronchiectasis (80). “Tramline” shadows and “finger-in-glove” opacities are temporary findings that indicate bronchial wall edema and thickening, whereas once the mucus plug is expectorated, it can remain as permanent parallel line shadows (5).

**Figure 2:**
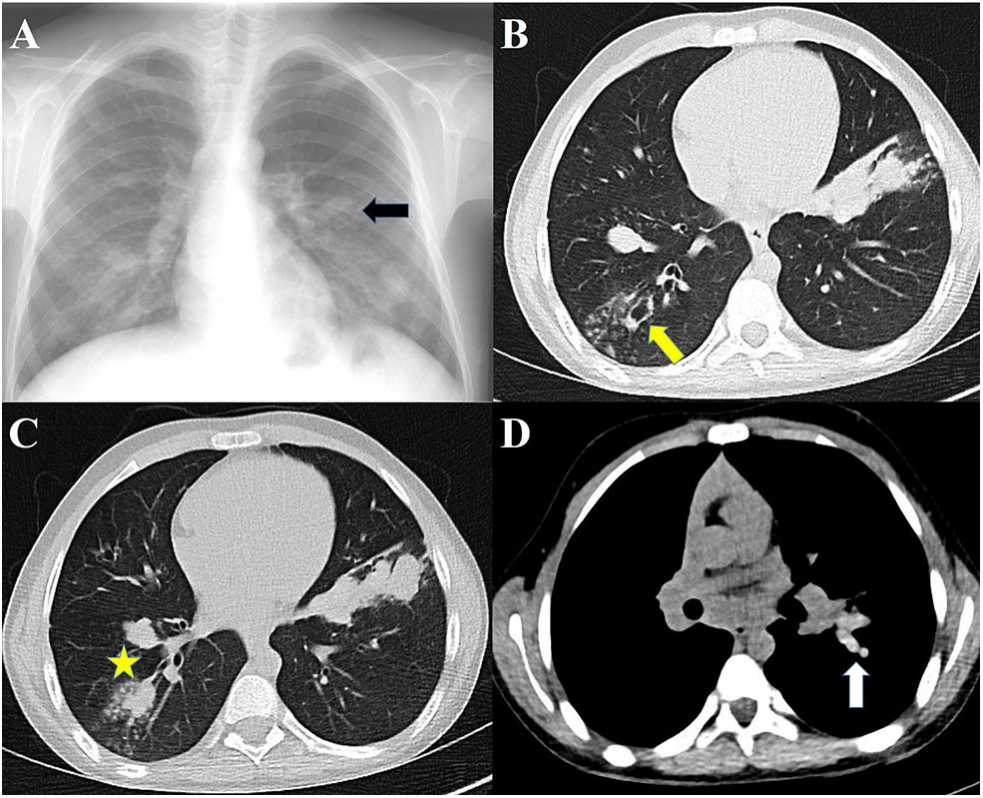
Radiographs of patients with ABPA. **(A)** Chest radiograph showing finger-in-glove sign, **(B)** HRCT showing central bronchiectasis, **(C)** HRCT showing mucus plugging in dilated bronchi, **(D)** HRCT showing high-attenuation mucus.

High-resolution CT (HRCT) of the lungs is more sensitive for detecting bronchiectasis distribution and other abnormalities that cannot be detected in chest radiography (40). The findings of ABPA on chest HRCT include centrilobular nodules, central bronchiectasis often with mucoid impaction “tree-in-bud” pattern, mosaic attenuation, fibrosis, and cavitation (81). Bronchiectasis seen in ABPA is typically central (bronchiectasis that involves two-thirds of the central part of the lung parenchyma) but peripheral bronchiectasis is not infrequent. Central or cystic varicose bronchiectasis, infiltrations that completely resolve with corticosteroid treatment, and mucus plugs are common in CF and ABPA, high-attenuation mucus (HAM) has been reported in 28% of patients with ABPA on HRCT. Mucoid impaction causes “toothpaste” shadows or “gloved-finger” shadows (75, 81, 82). HAM is the name given to mucus that appears denser than skeletal muscles (radiodensity of >70 Hounsfield units). Mucus plugs may cause segmental, lobar or total atelectasis. HAM and bronchiectasis are indicators of serious disease and recurrent exacerbations. Also, the presence of bronchiectasis makes it difficult to enter remission (83). In the later stages, pneumothorax might be seen in patients who develop pulmonary fibrosis (84). Pleural thickening is seen in ABPA and in advanced CF (85, 86). Radiographic findings in ABPA are demonstrated in Figure 2.

Chest HRCT findings in ABPA may correlate with the immunologic severity. Agarwal et al. reported that the presence of HAM plugs was more consistent with serologic severity and recurrent relapses (75, 83). ABPA can be classified radiologically based on clinical and HRCT findings (83) (Table 5).

**Table 5:** Radiologic classification of ABPA based on clinical and HRCT findings.

ABPA-S (Serologic ABPA)	ABPA without any radiologic findings on HRCT of the thorax
ABPA-B (ABPA- Bronchiectasis)	ABPA including bronchiectasis on chest HRCT
ABPA-HAM (ABPA- High-attenuation mucus)	ABPA including HAM on chest HRCT
ABPA-CPF (ABPA- Chronic pleuropulmonary fibrosis)	ABPA with two or more radiologic features suggestive of fibrosis (including fibrocavitary lesions, pulmonary fibrosis, pleural thickening) without the presence of mucoid impaction (or HAM)

*ABPA, allergic bronchopulmonary aspergillosis, HAM, high-attenuation mucus*.

Magnetic resonance imaging (MRI) has not been traditionally used for the evaluation of lung parenchyma due to the low proton density of the tissue and high susceptibility of artifacts. Recently with the major technological advancements; MRI has been used in the diagnosis of respiratory pathologies including ABPA. In the diagnosis of ABPA; MRI demonstrated high specificity and positive predictive value; but less sensitivity and negative predictive value compared with the HRCT scan in children (87). The match of HAM on MRI seems to be inverted mucus impaction, which is characterized by a high signal intensity on T1-weighted images and low signal intensity on T2-weighted images (87, 88). However; MRI is not being used as a routine clinical practice of patients with ABPA and there is a need for new clinical investigations to use MRI in the diagnosis of ABPA.

The differences and similarities of clinical features and diagnostic criteria of ABPA in patients with CF or asthma are summarized in Table 6.

**Table 6:** Similarities and differences of ABPA in patients with cystic fibrosis or asthma.

	**ABPA in CF**	**ABPA in Asthma**
Childhood onset	Common	Uncommon
Sex	Male/Female=1	Male/Female=1
Clinic	Pulmonary exacerbation of CF	Worsening of asthmatic symptoms
Mucus Production	Increased, brown-black	New, brown-black
Clubbing	Common	Rare
Eosinophilia	Uncommon	Common
Total IgE >1,000 IU/mL	+	+
Specific serologic test	rAsp f6 specific IgE	Combination of rAsp f4 and f6 specific IgE
Aspergillus skin prick test	+	+
Concomitant bacterial infections	Common	Uncommon
Bronchiectasis	Central, but generally extensive	Central
Transient pulmonary opacities	+	+
High attenuation mucus plugs on chest CT	+	+

*ABPA, allergic bronchopulmonary aspergillosis; CF, cystic fibrosis; IgE, immunoglobulin E; rAsp, recombinant Aspergillus fumigatus antigens*.

## Treatment

The principles in the treatment of ABPA include the use of anti-inflammatory agents to suppress the immune hyperreactivity and the use of antifungal agents to attenuate immune hyperresponsiveness by reducing the fungal burden in the airways (89). There are several goals of treatment such as treating the acute stage of ABPA, controlling symptoms of CF, preventing or treating pulmonary exacerbations of ABPA, and reducing progression to end-stage disease. Inadequate and delayed treatment can lead to complications such as pulmonary fibrosis, bronchiectasis, and loss of lung function (90). At the same time, treatment options should also have minimal or no adverse reactions.

Treatment of ABPA in CF is not much different from ABPA in asthma, and involves the use of corticosteroids, antifungal agents, and monoclonal antibodies, mainly prednisolone, itraconazole, and omalizumab, respectively. The doses, side effects and important comments of administration of the drugs used in the treatment of ABPA are summarized in the Table 7.

**Table 7:** Summary of drugs for children with ABPA in CF.

**Drugs**	**Dose**	**Adverse effects**	**Comments**
**Corticosteroid**			Systemic corticosteroids remain the mainstay of treatment
Prednisolone	Recommendation: Initial dose: 0.5–2.0 mg·kg^−1^·day^−1^ (max 60 mg) for 1–2 weeks, then 0.5–2.0 mg·kg^−1^·day^−1^ every other day for 1–2 weeks, then taper in next 2–3 months	Growth retardation, diabetes, hypertension, cataracts, acne, osteoporosis, increased appetite, weight gain, striae, susceptibility to infections, increased intracranial pressure, ulcer disease	Orally used prednisolone is the most recommended corticosteroid treatment model
	Alternative option: 0–2. weeks: 1 mg·kg^−1^·day^−1^ (max daily dose 50 mg) 2–4. weeks: 0.5 mg·kg^−1^·day^−1^ 4–6. weeks: 0.5 mg·kg^−1^ 3 times weekly 6–8. weeks: 0.25 mg·kg^−1^ 3 times weekly 8–10. weeks: 0.1 mg·kg^−1^ 3 times weekly		
Pulse steroid	10–20 mg·kg^−1^·day^−1^ intravenous for 3 days every 3–4 weeks for 6–12 months	Hot flashes, epigastric pain, headache, be aware of circulatory collapse following rapid administration of large doses of methylprednisolone	Long-term follow-up data are not available and this published experience was uncontrolled
Antifungal			Antifungal therapy has been used as an adjunct in the treatment of ABPA
Itraconazole	Recommendation: 5 mg·kg^−1^·day^−1^ once or twice a day (max 400 mg·day^−1^), for 3–6 months	Nausea, vomiting, hypokalemia, hepatotoxicity	First-line antifungal agent Liver function tests should be obtained at baseline, 1 month, and for every 3 months thereafter, or if there is a suspicion of liver dysfunction
Voriconazole	Dosage based on an uncontrolled, open label, retrospective review of children with CF and ABPA: • <12 years: 6 mg·kg^−1^ (max 200 mg) BD orally for 1 day, then 4 mg·kg^−1^ (max 100 mg) BD >12 years and <40 kg: 200 mg BD orally for 1 day, then 100 mg BD; • >12 years and >40 kg: 400 BD orally for 1 day, then 200 mg BD for a median of 22 weeks	Visual changes, photosensitivity, hepatotoxicity	The safety in children under the age of 12 has not been established. Dosage is based on observational study, no RCT Second line antifungal agent for patients who have not responded to or cannot tolerate itraconazole
	Dosage based on prescribing recommendation for invasive aspergillosis: >12 years: 6 mg·kg^−1^ BD for 1 day, 4 mg·kg^−1^ BD intravenous or 200 mg BD orally (<40 kg orally maintenance dose: 100–150 mg BD)		
Posaconazole	One dosage option based on a prospective, non-randomized, open-label observational study of children with CF and aspergillus- related lung disease: • >35 kg: 400 mg BD (liquid suspension) or 300 mg daily (tablets with a loading dose of BD on day 1) • >25 kg and <35 kg: 300 mg (liquid suspension) BD • <25 kg 18–24 mg·kg^−1^·day^−1^ BD for 12 weeks	Abdominal pain, nausea-vomiting, diarrhea, rash, fever, headache Hepatotoxicity QTc interval prolongation	The tolerability and efficacy in children under the age of 13 has not been established May be third line antifungal agent for patients who did not tolerate itraconazole and voriconazole Delayed-release tablets and oral suspension are not interchangeable due to the differences in the dosing of each formulation
	Another dosage option based on a case study of a children with CF and ABPA: 200 mg orally thrice per day		
Isavuconazole	Dosage based on case series of hemato-oncologic children (3–18 years) with invasive aspergillosis or mucormycosis and European Congress of Clinical Microbiology and Infectious Diseases 2018: 2–17 years: 10 mg·kg^−1^ (max 200 mg) every 8 h for the first 48 h, then 200 mg once daily (oral or intravenous) for a median of 75 days	Nausea, vomiting	Efficacy and safety have not been tested in children (<18 years) and the dosage and schedule have not been established. There is no published use in children with CF and ABPA and needs more studies, may be a rescue treatment
**Human monoclonal antibody**			
Omalizumab	Dosage based on case reports in CF children with ABPA: 300–375 mg SC every 4 weeks for 6–18 months Dosage based on prescribing recommendation for allergic asthma: 75 mg to 375 mg (determined by total Ig E and body weight) SC every 2–4 weeks	Mild rash, joint pain, bone fractures, nausea, dizziness, cold symptoms such as stuffy nose, sneezing, cough, sore throat	No RCTs evaluating the efficacy and safety profile of omalizumab in children with CF Approved for patients with severe asthma aged 6 years and older Early initiation of omalizumab may be an alternative therapy in patients with CF and ABPA in those who fail to respond to systemic corticosteroids or have severe adverse effects of prednisolone
Mepolizumab	Dosage based on a multinational, nonrandomized, open-label study of 6–11-year-old children with severe asthma: • <40 kg: 40 mg SC every 4 weeks • ≥40 kg: 100 mg SC every 4 weeks for 52 weeks	Headache, feeling tired, pain, swelling, redness, burning, or itching where the medicine was injected	There are case reports in adult patients with ABPA in CF There is no study for children with ABPA in CF Approved for patients with severe asthma aged 6 years and older
	Dosage based on prescribing recommendation for allergic asthma: • 6–11 years: 40 mg SC every 4 weeks • >12 years: 100 mg SC every 4 weeks		
Benralizumab	Dosage based on two phase-3 studies of 12–75-year-old patients with severe asthma: 12 years and >40 kg: 30 mg SC every 4 or 8 weeks (first three doses every 4 weeks) for 48 weeks	Headache, sore throat, fever, hypersensitivity reactions, injection site reactions (pain, redness, itching, or a small lump)	There are case reports in adult patients with ABPA in CF Approved for the treatment of severe asthma for 12 years and older
	Dosage based on prescribing recommendation for allergic asthma: >12 years: 30 mg SC every 4 weeks for the first 3 doses, and then once every 8 weeks		
Dupilumab	Dosage based on two phase-3 studies of >12-year-old patients with severe asthma: >12 years: 200–300 mg SC (loading dose 400–600 mg) every 2 weeks for 52 weeks Dosage based on prescribing recommendation for severe atopic dermatitis: >12 years: initial dose of 600 mg SC (two 300 mg injections), followed by 300 mg given every other week	Injection site reactions (erythema, edema), conjunctivitis, eye irritation, headache, herpes simplex viral infections	There are case reports in adult patients with ABPA in CF Approved for the treatment of moderate-to-severe atopic dermatitis and severe asthma for 12 years and older

*ABPA, allergic bronchopulmonary aspergillosis; BD, bis in die (twice a day); CF, cystic fibrosis; max, maximum; RCT, randomized controlled trial; SC, subcutaneously*.

### Corticosteroids

#### Oral Corticosteroids

Systemic corticosteroids are currently the most effective agents in the treatment of ABPA. Use of corticosteroids is based on clinical experience because randomized trials do not exist and are unlikely to be performed due to ethical concerns. Prednisolone is the most widely used corticosteroid, and the dosage and duration of treatment have been investigated in several trials.

The Cystic Fibrosis Foundation Consensus Conference recommends 0.5–2.0 mg·kg^−1^·day^−1^ prednisone equivalent (maximum 60 mg·day^−1^) for 1–2 weeks, then 0.5–2.0 mg·kg^−1^·day^−1^ prednisone equivalent every other day for 1–2 weeks, then tapering on the basis of clinical and immunologic improvement. An attempt should be made to begin to taper off corticosteroids in 2–3 months (3).

In patients with asthma with ABPA, Agarwal et al. compared two steroid regimens in a randomized controlled trial (RCT). The medium-dose regimen (0.5 mg·kg^−1^·day^−1^ for 1–2 weeks, then on alternate days for 6–8 weeks, taper by 5–10 mg every 2 weeks, and discontinue after 3–5 months) was equally efficacious as a high-dose regimen (0.75 mg·kg^−1^·day^−1^ for 6 weeks, 0.5 mg·kg^−1^·day^−1^ for 6 weeks, taper by 5 mg every 6 weeks to complete a total duration of 6–12 months) in terms of the improvement in lung function, time to first exacerbation, the number of subjects with exacerbation at 1 year, and the occurrence of corticosteroid-dependent ABPA at 2 years (91).

A third dose regimen of prednisolone different from the other two above (0.5 mg·kg^−1^·day^−1^ for 4 weeks, 0.25 mg·kg^−1^·day^−1^ for 4 weeks, 0.125 mg·kg^−1^·day^−1^ for 4 weeks, then taper by 5 mg every 2 weeks and discontinue after 4 months) was associated with 100% early composite response at 6 weeks (clinical, immunologic, and radiologic improvement) in three studies of patients with asthma with ABPA (92–94). Therefore, the third regimen may offer the right balance between early treatment response and toxicity.

The main goal in these studies was to investigate the treatment protocol with fewer adverse effects while providing similar efficacy. In order to reduce the adverse effects of steroid therapy, the addition of an antifungal agent to the treatment was investigated. Very recently, a study was performed to see the effectiveness of combining short-term prednisone (2 mg·kg^−1^·day^−1^ for 3 days, taper every 5 days to 1, 0.5, and 0.25 mg·kg^−1^·day^−1^ and discontinued after 18 days in total) and long-term itraconazole (10 mg·kg^−1^·day^−1^ for capsules and 5 mg·kg^−1^·day^−1^ for suspension for at least 12 months) in treatment of patients with CF and ABPA. It was shown that a combination of itraconazole with short-term prednisone improved long-term pulmonary outcome in patients with ABPA without undesired glucocorticoid adverse effects (95). In a recent survey, although the majority of consultants were found to use both corticosteroids and itraconazole to treat a first diagnosis of ABPA, only one-third was reported to use prednisolone alone (96).

#### Pulse Steroid Therapy

The role of intravenous (iv) corticosteroids in ABPA, especially “pulse” steroid therapy is still being investigated. Pulse steroid therapy consists of iv methylprednisolone infused daily for three consecutive days every month. Intravenous pulse steroid therapy in ABPA has been used in patients who have adverse effects with daily corticosteroids or do not respond to standard doses of oral steroid therapy, usually associated with prolonged use of steroids. However, there are no controlled trials comparing oral steroids with iv steroids. In several reports, pulse methylprednisolone was successfully used in oral steroid-dependent patients with CF and ABPA (10–20 mg·kg^−1^·day^−1^ for three consecutive days every month) (97, 98). In another report of an 11-year-old child with CF who was unresponsive to oral steroids, the use of iv pulse methylprednisolone made an improvement in clinical stabilization and better control of ABPA (20 mg·kg^−1^ for 3 days followed by 10 mg·kg^−1^ for 3 days) (99). In most of the studies, iv pulse steroid therapy was well tolerated and patients were able to stop the pulse therapy after 6–12 months with disease control (100).

#### Inhaled Corticosteroids (ICS)

ICS are known to have significantly fewer adverse effects compared with oral corticosteroids. Several case reports and small case series suggest some benefit of ICS in the management of ABPA without CF, but a study in 32 patients of ABPA found no benefit of using low doses of ICS (400 μg of beclomethasone per day) compared with placebo (101). In another study conducted retrospectively, 21 adult patients with asthma and serologic ABPA who refused conventional treatment received higher doses of ICS (1,600 μg of budesonide per day). The authors found that ICS were ineffective in controlling the immunologic activity because the total IgE levels continued to increase (102). As a result, ICS alone have no role as first-line therapy in ABPA.

### Antifungal Therapy

Antifungal azoles are the most frequently combined agents with steroids. It is frequently used in steroid-resistant cases or for the purpose of reducing steroid dose and duration (103). Antifungal agents are thought to decrease the fungal burden in the respiratory tract, hence reducing the antigenic stimulus responsible for the inflammation, improving symptoms, and possibly slowing progression (104). Thus, antifungal drugs can act as steroid-sparing agents.

#### Itraconazole

The most widely used azole in the management of ABPA is itraconazole. Although azoles are considered to be fungostatic drugs, itraconazole seems to be efficacious in the treatment because fungal burden in ABPA is thought to be lower than in other invasive disorders of fungi (27). Studies on treatment for ABPA in CF are outdated and contain few patients. Recently, two RCTs evaluated the role of itraconazole, but only adult patients with asthma with ABPA were included. In a study involving 55 patients who were using oral corticosteroids regularly, the subjects were randomized to receive itraconazole and placebo for 16 weeks. It was shown that the rate of response to therapy was significantly higher in the itraconazole group than in the placebo group (105). The other study included 29 patients with ABPA randomized to receive itraconazole or placebo for 16 weeks. In this study, itraconazole was found to be effective in normalizing eosinophilic airway inflammation, reducing systemic immune activation, and reducing severe exacerbations (106).

In an RCT, 131 adult patients with asthma with ABPA were randomized to receive either oral itraconazole or prednisolone. All subjects treated with prednisolone showed a composite response after 6 weeks of treatment, whereas 12% of subjects in the itraconazole group did not exhibit a composite response. All subjects who failed to respond to itraconazole were treated with prednisolone, and showed a composite response after 6 weeks of treatment. This study suggests that oral corticosteroids are more effective than itraconazole (100 vs. 88%) in the treatment of acute-stage ABPA (93). The Cystic Fibrosis Foundation Consensus Conference recommends that the initial dose should be 5 mg·kg^−1^·day^−1^, which may be given once or twice daily (maximum 200 mg·dose^−1^). The daily dosage should not exceed 400 mg·day^−1^ unless low serum itraconazole levels are obtained. The duration of therapy should be short (3–6 months) because of the emerging risk of azole-resistant Aspergillus species. In addition, itraconazole cannot be recommended for initial therapy in patients with CF and ABPA. However, it should be added to therapy if there is a slow or poor response to corticosteroids, for relapse of ABPA, in corticosteroid toxicity, and corticosteroid-dependent ABPA. For patients receiving itraconazole, liver function tests should be obtained before therapy. Routine liver function testing after 1 month and every 3–6 months thereafter should be considered. There are several medications that are known to interact with itraconazole. Therefore, determining serum concentrations of other drugs and/or itraconazole may be required. Itraconazole concentrations should also be determined when there is a lack of clinical response or if there is concern about adequate drug absorption or patient compliance. Besides, itraconazole is associated with gastrointestinal symptoms, congestive heart failure, and rash (3).

#### Newer Azoles

Few studies have evaluated the newer azoles (voriconazole, posaconazole and isavuconazole) for their efficacy in ABPA. In the largest study, Chishimba et al. retrospectively analyzed the efficacy and safety of voriconazole and posaconazole in 20 adult patients with asthma with ABPA. Overall, clinical improvement with voriconazole or posaconazole therapy was seen in about 70–75%, so both drugs were found to be alternative treatments to itraconazole (107). Monotherapy of voriconazole vs. prednisolone in patients with asthma with ABPA was evaluated in an RCT (92). Fifty subjects were randomized to receive either prednisolone or voriconazole. In this study, voriconazole monotherapy had similar efficacy to prednisolone. According to this study, voriconazole appeared to be as effective as corticosteroids in acute-stage ABPA. Fewer studies have been conducted on voriconazole therapy in patients with CF and ABPA. Glackin et al. reported that serum total IgE level was decreased with voriconazole therapy in patients with CF (108). In the other study, an uncontrolled, retrospective study in 21 patients with ABPA in CF showed an improvement in lung function with voriconazole therapy (109). Unique adverse reactions among patients receiving voriconazole include transient vision changes, visual hallucinations, and photosensitivity. However, voriconazole is a reasonable alternative to itraconazole because it is better tolerated in some patients and is well-absorbed.

In a retrospective study that compared posaconazole with other azoles in the treatment of ABPA in 32 adult patients with CF, it was found that there was a significant reduction in specific IgE to Aspergillus with posaconazole compared with itraconazole and voriconazole (110). Recently, Patel et al. reported a prospective single-center, non-randomized, open-label observational study over a 53-months period evaluating the safety, tolerability, and efficacy of posaconazole in pediatric patients with CF. A total of 23 episodes of Aspergillus-related lung disease were treated. Posaconazole was well-tolerated in children with CF and an improvement in FEV1 and serologic parameters in response to posaconazole was noted in this study (111). It is also associated with gastrointestinal symptoms depending on the formulation, and there are sparse data supporting its use for ABPA.

Isavuconazole is also a new azole that is approved for primary therapy of invasive aspergillosis. However, the use of isavuconazole in ABPA is less well-studied. The first report of the use of isavuconazole for the treatment of ABPA presented an adult patient with asthma who was successfully treated with isavuconazole after unsuccessful treatment with corticosteroids, itraconazole, and voriconazole (112). The patient tolerated isavuconazole well, had marked symptomatic improvement, and demonstrated a normal FEV1/FVC ratio for the first time in 7 years after being diagnosed as having ABPA. Treatment with isavuconazole is generally safe and well-tolerated but there are no studies on isavuconazole treatment of ABPA either in the pediatric population or patients with CF. However, a non-randomized open-label multicenter study on isavuconazole treatment of invasive aspergillosis or invasive mucormycosis in pediatric subjects is underway and planned to be completed in 2 years (ClinicalTrials.gov; NCT03816176).

#### Nebulized Amphotericin B (NAB)

NAB is an option for maintaining remission in recurrent exacerbations. In one RCT (21 adults with asthma), non-liposomal NAB without concomitant azole therapy was found to be efficacious in maintaining remission in those with recurrent exacerbations. On the other hand, NAB had poor efficacy in inducing a response in patients with acute-stage ABPA or during an exacerbation of ABPA (113).

In a case report of a pediatric patient with end-stage CF lung disease with progressing symptoms, very poor lung function and severe bronchiectasis, treatment with NAB resulted in improvement of cough, dyspnea, hypoxia, 6-min walk test, reduction in oral corticosteroid dosages, and pulmonary function with no adverse events (114).

NAB has the potential to precipitate or worsen bronchospasm, especially the deoxycholate preparation; therefore, the first dose should be administered with caution and short-acting bronchodilator may be administered 15–30 min prior to NAB (113).

### Monoclonal Antibodies

Corticosteroids can control the symptoms of most patients and their combination with antifungal drugs increases treatment success. However, some patients become steroid-dependent or experience adverse effects. In an effort to treat refractory patients or to reduce adverse effects, many physicians have tried monoclonal antibody treatment. As a monoclonal antibody, although omalizumab is the most preferred, mepolizumab and benralizumab are the most investigated.

#### Omalizumab

Omalizumab is a humanized monoclonal antibody against IgE recommended for the treatment of uncontrolled allergic asthma and chronic spontaneous urticaria. It is considered for use in the treatment of other allergic disorders such as ABPA because its mechanism of action is via IgE antagonism. Although omalizumab seems to facilitate ABPA control in asthma, evidence for use in patients with CF is currently limited to data from case reports. In addition, an RCT evaluating the safety and efficacy of omalizumab for the treatment of ABPA in patients with CF aged 12 years and older was designed, but this study was terminated prematurely due to adverse events (115). Another RCT was reported in 2015 which evaluated the clinical and immunologic effects of omalizumab in asthmatic ABPA patients. Thirteen patients with chronic ABPA were randomized to a 4-months treatment with omalizumab or a placebo followed by a 3-months washout period. The ABPA exacerbations were significantly less frequent during the active treatment phase compared with the placebo period (116). Van der Ent et al. reported the first case of a single dose of omalizumab treatment in a 12- year-old girl with CF and ABPA who showed a rapid and good improvement of clinical signs and lung functions (117). In a case series of Emiralioglu et al. six patients with CF and ABPA who had received omalizumab were reported. Omalizumab (300 mg dosage) was administered subcutaneously every 4 weeks to the patients who were treated previously with oral prednisolone and itraconazole. Decreased IgE levels, improvement in respiratory symptoms, and a steroid-sparing effect was shown with omalizumab treatment (118). A retrospective multicenter observational French study retrieved 32 patients with ABPA and CF (11 children and 21 adults) who had received omalizumab for more than 3 months. Among them, 14 patients were able to discontinue steroid treatment or reduce their daily dose during follow-up (119). Very recently, a retrospective study of 27 adult CF patients receiving omalizumab for asthma or ABPA was conducted by Koutsokera et al. to evaluate the efficacy and safety of treatment. Omalizumab was found to be effective in the improvement of respiratory functions in adult CF patients with difficult-to-control asthma or ABPA with no significant adverse effects during the study period (120).

Furthermore, a standard dose has not been established for omalizumab in patients with CF and ABPA. Different studies used various doses (ranging from 225 mg to 750 mg) and frequency of treatment (ranging from once per week to once monthly) according to the weight and serum IgE level. However, the most commonly used regimen was 375 mg every 2 weeks (121). The duration of treatment is also controversial. As an example, Wong et al. have reported 2 patients with CF and steroid-dependent ABPA who were successfully able to be weaned off steroid therapy with the treatment of omalizumab monthly for 2 years (122).

In contrast to the above case reports, two studies on patients with CF with ABPA found no benefit with omalizumab treatment. In one of them, Brinkman et al., reported a 15-year-old patient who could not be weaned from steroid therapy for over 12 months with omalizumab treatment (123). Ashkenazi et al. also presented nine patients with CF and ABPA who were treated with 300–375 mg omalizumab every month but did not respond to treatment (124). However, in both studies, they administered the drug every month with a lower dose compared with other cases.

Omalizumab may be a promising treatment option also for CF patients with chronic bacterial infections since use of corticosteroids is of concern due to compromised immunity for these patients. In a recent retrospective cross-sectional study, no significant adverse events or worsening of infection due to omalizumab treatment were observed in patients with ABPA and chronic bacterial airway infection. Consequently, treatment with omalizumab was found to be effective and safe in patients with ABPA, regardless of concurrent chronic respiratory tract infections since it does not exhibit immonusuppressive effects (125).

#### Mepolizumab

Mepolizumab is an anti-IL-5 monoclonal antibody used for severe refractory eosinophilic asthma. Altman et al. were first to demonstrate mepolizumab as an additional and effective treatment option for severe ABPA resistant to corticosteroids, antifungal therapy, and omalizumab in a 58-year-old asthmatic woman (126). In this case, clinical improvement was achieved with the addition of 100 mg mepolizumab every 4 weeks to high-dose omalizumab treatment.

In another case, mepolizumab was shown to be effective as a monotherapy in a 64-year-old woman treated for severe bronchial asthma with ABPA exacerbation (127). In this case, after a successful 3-years treatment period with systemic corticosteroids and itraconazole, deterioration occurred. With the addition of 100 mg mepolizumab every 4 weeks, dramatic improvements were observed in symptoms, lung function, peripheral eosinophil counts, and chest imaging.

#### Benralizumab

Benralizumab is a monoclonal antibody directed against the alpha chain of the IL-5 receptor. Recently, two adult cases of ABPA with asthma were reported to switch to benralizumab after treatment with mepolizumab (128). Benralizumab is thought to clear bronchial mucus plugs, prevent irreversible airway damage, and improve the prognosis of patients with ABPA.

#### Dupilumab

Dupilumab is a humanized monoclonal antibody. It is a dual inhibitor of IL-4 and IL-13 pathways. In a case series, the clinical course of three adult subjects with asthma and ABPA treated with dupilumab over 6 months was presented (129). In this study, dupilumab was found to facilitate disease control in ABPA, with reduced symptoms and oral corticosteroid use.

### Treatment Practices

In acute ABPA, systemic corticosteroids are the first choice of treatment. Although any of corticosteroid regimens as detailed above can be used, high-dose steroids should not be preferred as the first choice in the management of ABPA due to the more frequent adverse effects. The steroid dose should be adjusted according to the clinical and immunologic characteristics of disease. Antifungal agents should be added to therapy if there is a slow or poor response to corticosteroids, for relapse of ABPA, in corticosteroid toxicity, and corticosteroid-dependent ABPA. Azole therapy is usually begun with itraconazole; newer azoles are reserved for those who fail therapy or experience adverse reactions with itraconazole or fail to achieve optimal serum levels of itraconazole, despite receiving the maximum dose (104). Nonetheless, a small number of patients may require chronic corticosteroid therapy (3).

After starting treatment for acute ABPA, monitoring is performed with clinical evaluation, serum total IgE levels, spirometry, and chest radiography. It is not helpful to measure Aspergillus-specific IgE and IgG during treatment because their levels are not correlated with the reduction in the serum total IgE or clinical or radiologic improvement. Serum total IgE concentrations should be measured every 6–8 weeks, especially in the first year (130). The clinical effectiveness of therapy is evaluated through serum total IgE levels. The goal of therapy is not to achieve normal IgE levels but to decrease its levels by 35–50% at 8 weeks, which leads to clinical and radiographic improvement. During the treatment, serum total IgE levels are measured to determine the new baseline IgE concentrations (5). The lowest value achieved after treatment is taken as the new baseline. An increasing level (>100% of the new baseline) of total IgE along with worsening respiratory symptoms and the appearance of consistent radiologic findings suggest an exacerbation of ABPA (131). Twenty to 35% of relapses are asymptomatic and are detected radiographically and serologically. The treatment of the first exacerbation is similar to the treatment of acute disease. Greater than or equal to two exacerbations within 6 months of stopping therapy or worsening of clinical and/or radiologic condition, along with immunologic worsening (rise in IgE levels) on tapering oral steroids/azoles is steroid-dependent ABPA. Pulse steroids may be considered in such patients. If they are currently taking itraconazole, newer azoles may be considered. Omalizumab has shown promise in such cases but its use in patients with ABPA and CF requires more definitive clinical trials. Furthermore, it is also very important to identify and exclude any potential environmental exposure source of *A. fumigatus* because it can initiate exacerbations.

Remission may be considered if the patient has remained asymptomatic with stable IgE levels (persisting at/below baseline or increase by <50%) for at least 6 months without the requirement of corticosteroid or antifungal therapy. In the remission period, monitoring may be performed every 3 months for a year and every 6 months thereafter with a clinical examination and serum total IgE levels. Chest radiography may be obtained if clinically indicated. Spirometry is performed in routine follow-ups and in response to changes in symptoms. Antifungal therapy is not used to prevent exacerbations given the potential toxicity and lack of proven benefit.

Another considerable point of management is that chronic respiratory tract infections are almost inevitable in ABPA patients with CF. These patients are especially vulnerable to *Pseudomonas aeruginosa* or *nontuberculous mycobacteria* because of the combined effects of structural deformities in the airways and compromised immunity caused by systemic and local administration of corticosteroids (132). In that case, monoclonal antibodies such as omalizumab may be a good choice since it can prevent the use or reduce the doses of systemic corticosteroids (125).

In summary, clinical improvement is generally achieved with proper diagnosis, follow-up, and treatment. At the same time, there is a considerable variation in treatment practices of patients with CF and ABPA, hence there is a pressing need for new guidance in both treatment and its duration.

## Author Contributions

BS, DA, BO, NE, EY, and UÖ have made contributions to the design, editing, and writing of this manuscript. All authors contributed to the article and approved the submitted version.

## Conflict of Interest

The authors declare that the research was conducted in the absence of any commercial or financial relationships that could be construed as a potential conflict of interest.
